# Disparities in risky sexual behavior among khat chewer and non- chewer college students in Southern Ethiopia: a comparative cross-sectional study

**DOI:** 10.1186/s12889-018-5405-x

**Published:** 2018-04-27

**Authors:** Eyasu Ware, Gurmesa Tura, Tsedach Alemu, Eshetu Andarge

**Affiliations:** 1 Department of Public Health Nursing and Health Extension, Arba Minch College of Health Sciences, P.O.Box: 155, Arba Minch, Ethiopia; 2 Department of Population and Family Health, Faculty of Public Health, Jimma University, P.O.Box:378, Jimma, Ethiopia; 3 Department of Reproductive Health and Nutrition, School of Public Health, College of Health Sciences and Medicine, Wolaita Sodo University, P.O.Box:138, Wolaita, Ethiopia

**Keywords:** Khat chewing, Risky sexual behavior, College students, South Ethiopia

## Abstract

**Background:**

Risky sexual behavior (RSB) and its consequence among school adolescents and youths have been well understood. It is still a common practice among college and university students living away from their controlling families compounded with the ever-worsening khat chewing habits. However, the relation between khat chewing and RSB is not well studied particularly among college students in Ethiopia. Hence, this study contributes to the literature by examining disparities of RSB among khat chewer and non-chewer students in Southern Ethiopia with the purpose of improving adolescent and youth health.

**Methods:**

An institution-based comparative cross-sectional study was conducted among 1211 college students at Arba Minch town in March 2015. Respondents were selected by employing a simple random sampling technique. Data was collected by using a pre-tested, structured, self- administered questionnaire. The data was entered into Epidata version 3.1 and analyzed using IBM SPSS statistics version 21. Level of statistical significance was declared at a *p*- value of < 0.05.

**Results:**

The prevalence of lifetime and current RSB among college students was 40.8 and 36.5% respectively. The lifetime and current prevalence of RSB among khat chewers (82.2 and 30.9%) was significantly higher than non-chewers (74.2 and 27.6%) respectively (*P*-value = 0.001). Male sex (AOR = 1.82; 95% CI = 1.28, 2.6), urban residence (AOR = 1.63,95% CI = 1.17, 2.28), age of students (AOR = 1.18; 95% CI = 1.09,1.28), living away from family (AOR = 2.45, 95% CI = 1.62,3.7), having high peer pressure (AOR = 2.58, 95% CI = 1.85–3.59), an increase in average grade point (AOR = 0.98, 95% CI = 0.96–0.99), regular attendance of religious institutions (AOR = 0.24, 95%CI = 0.12, 0.42), watching pornographic movies (AOR = 2.51, 95% CI = 1.79,3.51), khat chewing (AOR = 3.02, 95% CI:=1.91,4.76) and alcohol drinking (AOR = 2.26, 95% CI = 1.54,3.35) were factors associated with RSB.

**Conclusions:**

Considerable proportions of students were engaged in khat chewing and RSB. RSB was significantly higher among khat chewers as compared to non- chewers. Comprehensive sexuality education was recommended to college communities and by extension to the ministry of health and education to address the identified factors so that RSB can be reshaped.

**Electronic supplementary material:**

The online version of this article (10.1186/s12889-018-5405-x) contains supplementary material, which is available to authorized users.

## Background

Risky sexual behaviors (RSB) are behaviors that increase the risk of a negative reproductive outcome [1]. RSB includes early initiation of sexual intercourse, having more than one sexual partner, changing sexual partners frequently, having oral, vaginal, or anal sexual contact without a condom, using unreliable methods of birth control, or using birth control methods inconsistently or engaged in sex with commercial sex workers [1, 2].

Risky sexual behavior was more common in countries with lack of access to quality reproductive health care services like Cambodia, Tanzania, and Ethiopia [3–5]. In Cambodia, out of sexually active high school students, 34.6% were having two or more sex partners and 52.6% did not use a condom during their last sexual intercourse [4]. In Tanzania, 30.3% secondary school students reported being sexually active within the year prior to data collection. Among them, 41.7% had multiple sexual partners, 10.5% had concurrent sexual partners, and 41.1% did not use a condom during the last sexual intercourse [5]. In Ethiopia, 50.7–60.9% college students had unsafe sexual practice in their lifetime. Out of sexually active college students, 69.0% had their first sexual intercourse under the age of 18, 74.1% had sex with more than one sexual partner in the last 12 month, 41.0% didn’t use condom or used it inconsistently in the first sexual intercourse and 11.4% had sex with commercial sex workers [6, 7].

**Fig. 1:**
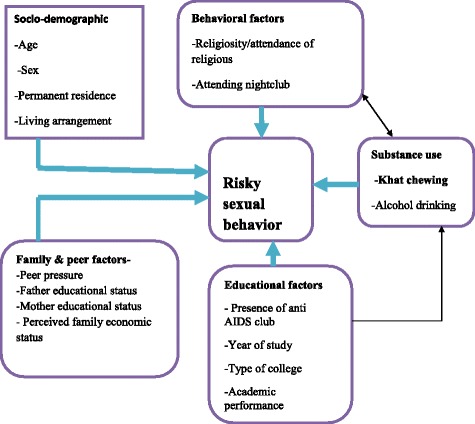
Conceptual framework of the study adapted from literatures

Khat consumption is one of the common problems among youths especially high school, college and university students [8]. A study from Yemen and Ethiopia showed that 54 and 19.6% of college students respectively chewed khat [9, 10]. The active constituent of khat releases nor-epinephrine that brings about increased sympathetic nervous system activities, which initiates sex drive and increases sexual arousal in women through increase in vaginal pulse amplitude and vaginal blood volume [11]. Khat is a psychotropic and mind-altering drug, its use could possibly alter rational decision-making and increase risk-taking behavior, and as a result it prompts risky sexual behavior [8]. Studies conducted among university students in Jimma, South West Ethiopia; high school students in Humera and college students in Bahir Dar, North West Ethiopia showed that RSB among chewers (51, 61.1 and 52.5%) was significantly higher than non-chewers (19.8, 9.3 and 16.5%) respectively [6, 12, 13]. A study from Haramaya University showed RSB was slightly higher (68.4%) among khat chewers than non-chewers (62.7%) [14]. The combined use of khat and other abusive substances showed an increase in the risk of RSB [15, 16]. The conceptual framework depicting the relationships between the variables of the study is shown below (Fig. 1).

Unlike governmental universities, governmental and non-governmental colleges in Ethiopia do not have accommodation for their students. Majority of the students come from rural areas and live in rental houses away from their supervising families and this could open a door for risky sexual practices and khat chewing [6, 10, 17]. Some students face financial problem to cover their basic needs. In the essence of fulfilling those needs, female students engage themselves in sexual relations with men to earn money in return. In South Ethiopia, out of the total sexually active female students, 22% took to sexual activity as a means of generating income though it was not as publicly as the bar ladies or prostitutes do [18].

Ethiopia is in a concerted effort to enhance safe sexual behavior of youths using different policies, strategies and activities. Reproductive health activities being within schools (primary, secondary and higher education institutions) include: school community conversation, peer education, life skill education, strengthening and supporting anti Acquired Immuno Deficiency Syndrome (AIDS) clubs and AIDS resource centers [19]. However, RSB and its consequences, Sexually Transmitted Infections (STIs) and unwanted pregnancy are growing concerns in educational settings. It claims the lives of the most productive segments of the society which in turn can lead to both immediate and long- term social and economic costs [3, 13, 20, 21]. In 2011, the self- reported STIs prevalence among Wolaita Sodo University students with the mean age of 20.7 ± 1.9 was 19.5%. It was seven-fold of the Ethiopian national rate of women and men in reproductive age group [20, 22]. About 8.5% of female college students were pregnant. Out of these, 76.9% was unwanted which leads to unsafe abortion and death [21]. These figures might be higher in Arba Minch town as it is one of the commonest tourist destination towns in Ethiopia with an estimated 128,025 tourists visiting the town in a year [23]. As reports from culture and tourism office of Gamo Goffa Zone indicate, sexual behavior and khat chewing habits of school adolescents and youths are altering due to tourists’ preference of young adolescents over commercial sex workers for sexual relations [24]. In Arba Minch hospital, 104 new Human Immuno Virus (HIV) positive cases were found among youths in the age range of 15–24 years and about 268 abortions were reported in 2014 [25]. Khat selling shops are increasing from time to time in the town. The increasing reports on negative reproductive health outcomes compounded with an increase in khat selling shops and khat chewing habits pressed us to assess the disparities in RSB among chewer and non-chewer college students and the factors associated with it in a college setting in Arba Minch town (South Ethiopia) where there is scarcity of evidence.

Moreover, previous studies in the country considered RSB as a composite measure of only having multiple sexual partner and exposure to unprotected sex without considering sex with commercial sex workers and early initiation of sexual practice. However, this study looks into RSB in a comprehensive way. Risky sexual behavior was measured as a composite variable of multiple sexual partner, sexual contact without condom, early initiation of sexual practice and sex with commercial sex worker owing to the fact that such types of comprehensive studies are limited in the country as well as in the study area [1, 2]. This study is mainly intended to compare the disparities in RSB among khat chewer and non-chewer college students by their socio-demographic and other behavioral characteristics and also to identify whether khat chewing status of the participants is an independent factor or not for RSB controlling for other socio-demographic and behavioral factors. The finding of this study is expected to fill the information gap to control negative reproductive health outcomes in the efforts to improve adolescents and youth health. Specifically, the finding is crucial for local planners & non-governmental organizations for improvements in their programmes.

## Methods

### Study setting and design

The study was conducted in Arba Minch town, Southern Ethiopia. The town is located 505 km to the South of Addis Ababa, the capital city of Ethiopia. According to data obtained from the town administration, there are three public and five private colleges in the town. In 2015, 8563 students were enrolled in the colleges in the regular programs (first year = 3604, second year = 2721 and third year =2238). Out of these, 4375 were male and 4188 were female [26]. In the year 2013/2014, more than 128,025 tourists visited the town. Currently, there is only one youth center in the town [23]. An institution- based comparative cross-sectional study was conducted among selected regular diploma level college students in Arba Minch town who were registered in 2014/15 academic year and available during the data collection period. Those students who were unable to see, hear and speak were excluded.

### Sample size and sampling procedure

The sample size was determined by using Epi info (7.1) statistical software using the following assumptions. The proportion of khat chewer college students who were sexually at risk = 50%, the proportion of non-chewer college students who were sexually at risk =40% [27], 95% confidence, 80% power, ratio of khat non-chewers to chewers(r) = 4. The sample size initially calculated using the above assumptions was 1264. After applying finite population correction formula and a 10% non-response rate, the final sample size was 1211.

The list of students was obtained from all the eight colleges and used as a sampling frame. Students were stratified based on their class years (first, second and third year). Sample was allocated for each strata based on proportional allocation to their size. Finally, students were selected from each stratum by random selection using SPSS software after preparing a sheet containing names of the students and their respective colleges.

### Data collection method, instrument and procedures

Data was collected by using a pre-tested, structured, and self -administered questionnaire. Pre-test of the questionnaire was conducted on 5% of the sample in Kemba Technical and Vocational Education and Training (TVET) college. The data was collected in a single working day by using six data collection facilitators and three public health professional supervisors. A day before the data collection, the randomly selected students were invited to participate in the study through posting their names on the notice board in their respective colleges. On the date of the data collection, the study participants were gathered in a hall in the morning session and they were informed about the objectives of the study. After obtaining a written consent and clarifying their right to decline from participation in the study, they were provided with the questionnaire. At the end, each respondent was allowed to put the completed questionnaires in the boxes ready in advance for collection.

### Data quality assurance

Training was given for all facilitators and supervisors. The questionnaire was translated from English language to Amharic language and to check for the original meaning, another person re-translated the Amharic version back to English. Sitting arrangement of the participants was organized and orientation for complete and accurate response was given prior to distribution of the questionnaire. Data were obtained from all selected participants in a college simultaneously and from the participants in all colleges in one day. The supervisors and the principal investigator monitored completeness of response on the questions carefully.

### Definitions and measurements

#### Socio-demographic characteristics

The participants were asked on their socio-demographic characteristics as categorical or continuous variables. Hence, information was obtained on sex (male/female), year of study (1st to 3rd),college type (public or private), religion (Orthodox/Protestant/Muslim/Others), marital status (single/married/divorced/others), place of origin (urban/rural/peri-urban), living arrangement (with family/friends/alone/other), father’s and mother’s highest educational level (no education/primary school/secondary school/higher education) as categorical variables. Information on participant’s age, Cumulative Grade Point Average (CGPA), families’ average monthly income was obtained directly as a continuous variable. Academic performance was measured based on student’s recent CGPA in the college as reported by him/her.

#### Khat chewing and other related behaviors

Respondents were asked on their chat chewing status and other related behaviors whether they ever (in 6 months before the survey date) chewed khat, smoked cigarette, drank alcohol, attended night club and anti-AIDS club, and watched pornographic movies or not. Chewers were further interviewed about when and where they started chewing khat, how long they chewed khat, reason for chewing, frequency of khat chewing per week, presence of khat chewing habit in their family. Nightclub and anti-AIDS club attendants and those who watched pornographic movies were further asked on their frequency of attendance.

A respondent was considered as lifetime khat chewer when he/she had ever chewed khat in his/her lifetime till the survey date while a current khat chewer was a respondent who had chewed khat in the last 6 months from the survey date. A habitual khat chewer was a respondent who had history of chewing khat for more than 3 days in a week while a long–time khat chewer had history of chewing khat for more than two years preceding the survey date.

#### Attendance to a religious institution

The study participants were asked on how often they go to a religious institution for worshiping purpose with the response options of number of days per week, 2 weeks period or rarely in more than two weeks’ time or not at all. A respondent was considered as a regular attendant when he/she visited a religious place (church, mosque etc.) at least twice in a week while an occasional attendant was a respondent who did not visit a religious place in two weeks but who has the habit of visiting religious place in more than 2 weeks period. A non-attendant was a respondent who did not ever visit a religious place.

#### Sexual behavior

The participants of the study were asked on their sexual behaviors whether they had ever had sexual intercourse or not (Yes = 1 or No = 0). The participants who reported that they had history of sexual intercourse (Yes = 1) were further asked the following questions; in their lifetime or in the 6 months before the survey date.

1. Age at first sex, number of sexual partners and reason for first sexual debut, reason for not using condom (condom non-users), and place they obtained the condom (condom users) as open-ended questions and
2. History of having sex with commercial sex workers, history of sex out of marriage for married participants, and use of condom in every act of sex or with a non-regular partner (married participants) as (Yes = 1 or No = 0) questions.

Risky sexual behavior of sexually active participants was measured by coding positive responses (Yes = 1) and negative responses (No = 0) to the following four variables related to risky sexual behavior. 1. Whether their age at first sex is less than 18 years or not [yes (less than 18) = 1),no(more than 18) = 0)], 2. Whether they used condom inconsistently with a non-regular partner or not [yes (inconsistent use) = 1), no (consistent use) = 0)], 3. Whether they had more than one partner or not [yes (more than one partner) = 1), no(a single partner = 0)], and 4. Whether they had a history of sexual intercourse with a sex worker or not [yes (had the history) = 1),no (had not had the history) = 0)]. RSB was computed in the SPSS using the compute command under the transform menu. Those respondents who replied ‘Yes’ to one of the above questions related to RSB (scored 1 or above on index for RSB) were categorized as having RSB. Those who replied ‘No’ to all of the questions related to RSB (scored 0 on the index for RSB) were categorized as having no RSB. The respondents reported on the above behaviors for both lifetime and in the 6 months before the survey date. A respondent was considered as having lifetime RSB when he/she had a RSB ever in his/her lifetime while he/she was considered as having current RSB when he/she had a RSB in the 6 months before the survey date.

Consistent condom use was defined as a respondent’s use of condom during every sexual encounter with a non-regular partner. Non-regular partner was a sexual partner out of marital union for a married participant. Peer pressure for risky sexual behavior was measured from three questions with yes/no options of answer 1. Whether a respondent’s best friend has started sexual intercourse or not 2. Whether there was initiation from the respondent’s best friend for his/her first sexual intercourse or not and 3. Whether majority of the respondent’s friends have sexual experience or not. Those respondents who replied yes to 2 of the 3 questions were considered as having high peer pressure for risky sexual behavior [28–30].

### Data analysis

Data was coded, checked and entered into Epidata version 3.1 and exported to IBM SPSS statistical software version 21 for cleaning and analysis. After cleaning data for inconsistencies and missing values in SPSS, descriptive statistics was done to show magnitudes of the variables using frequencies and percentages. Comparison of risky sexual behaviors among khat chewers and non-chewers was made using the Pearson chi-square test statistics in the SPSS to check whether risky sexual behavior of the students had an independent relationship with their chewing status. Bivariate logistic regression analysis was done and all explanatory variables that have association with the outcome variable at *p*-value < 0.25 were selected for multi-variable logistic regression analysis. Hence, the following variables had an association with current RSB at *p* value of less than 0.25. The respondent’s age, sex, place of residence, living arrangement, year of study, CGPA, frequency of attendance of religious institution, attending night club, watching pornographic movies, khat chewing status, alcohol drinking, cigarette smoking status, father’s educational status, peer pressure and type of college. However, mother’s educational status, perceived family economic status and participation in anti-AIDS club did not show an association with the outcome variable at *p* value of less than 0.25. In addition to this criterion, public health or clinical importance of variables was also considered. Hence, variables with prior strong significance (frequency of attendance of religious institution, attending night club, watching pornographic movies, khat chewing status, alcohol drinking, cigarette smoking status, and peer pressure) in previous literatures was considered regardless of the *p*-value consideration. Multicollinearity was checked by looking in to variance inflation factor (VIF) value (> 10) from a multiple linear regression model and standard error of greater than 2 in the final model in logistic regression to exclude the variable from analysis. Model fitness was checked by using Hosmer and Lemeshow goodness of fitness test as poor fit is indicated by a significance value less than 0.05. Then multi-variable logistic regression analysis using enter method was done and level of significance was declared at a *p* value of < 0.05 and AOR with its 95% CI was used to measure the degree of association between independent variables and the outcome variable. In the final model: participant’s age, sex, place of residence, average grade, living arrangement, peer pressure, attendance to religious institution, watching pornographic movies, khat chewing and alcohol drinking status showed significant association with current RSB.

### Ethics approval and consent to participate

Ethical clearance was obtained from the Ethical Review Committee of Jimma University. An official letter of co-operation was taken to the respective colleges. Participation was voluntary and written informed consent was obtained from each participant. The names of respondents and other identifiers (student-identity, College ID number etc.) were not included in the questionnaire to keep the confidentiality of the participant’s information.

## Results

### Socio-demographic and behavioral characteristics of the respondents by their khat chewing status

A total of 1109 students participated in the study, making a response rate of 91.6%. One hundred thirty seven (73.7%) of chewer and 450 (48.8%) of non-chewer respondents were males. The mean age of chewers and non-chewers was 20 ± 2.25 and 19.8 ± 1.93 respectively. Regarding year of study, 58 (31.2%) of chewers and 405 (43.9%) of non-chewers were first year students. Forty-five (24.2%) of khat chewer and seven (0.8%) of non-chewer students reported that they were smoking cigarette. Similarly, 145 (78%) of khat-chewers and 178 (19.3%) of non-chewers drank alcohol in the last six months. Fourteen (7.5%) and 272 (7.8%) of khat-chewers and non-chewers regularly participated in anti-AIDS club respectively. Thirty-four (18.3%) of khat-chewers and 310 (33.6%) non-chewers attended their religious institutions (churches, mosques etc.) regularly Almost six in ten (60.8%) of chewers and 121 (13.1%) non-chewers attended nightclubs. Majority (82.8%) of chewers and 425 (46%) non-chewers watched pornographic movies six months before the survey time (Table 1).

**Table 1:** Socio-demographic and behavioral characteristics of college students in Arba Minch Town, March 2015

Variables (*N* = 1109)		Total	Life time khat chewing status	
			Chewer	Non chewer
		No (%)	No (%)	No (%)
Sex	Male	587(52.9)	137(73.7)	450(48.8)
	Female	522(47.1)	49(26.3)	473(51.2)
Permanent residence	Rural	569(51.3)	56(30.1)	513(55.6)
	Urban	540(48.7)	130(69.9)	410(44.4)
Living arrangement	Away from family	852(76.8)	143(76.9)	709(76.8)
	With family	257(23.2)	43(23.1)	214(23.2)
Year of study	1st	463(41.7)	58(31.2)	405(43.9)
	2nd	357(32.2)	65(34.9)	292(31.6)
	3rd	289(26.1)	63(33.9)	226(24.5)
Type of college	Public	930(83.9)	151(81.2)	779(84.4)
	Private	179(16.1)	35(18.8)	144(15.6)
Mother’s educational status	No formal education	395(35.6)	51(27.4)	344(37.3)
	Primary	402(36.3)	60(32.3)	342(37.1)
	Secondary & above	312(28.1)	75(40.3)	237(25.7)
Father’s educational status	No formal education	221(19.9)	18(9.7)	203(22)
	Primary	404(36.4)	57(30.6)	347(37.6)
	Secondary & above	484(43.7)	111(59.7)	373(40.4)
Cigarette smoking	Yes	52(4.7)	45(24.2)	7(0.8)
	No	1057(95.3)	141(75.8)	916(99.2)
Alcohol consumption/drinking	Yes	323(29.1)	145(78)	178(19.3)
	No	786(70.9)	41(22)	745(80.7)
Participation in anti- AIDS club	Regularly	86(7.7)	14(7.5)	72(7.8)
	Often	153(14)	9(4.8)	144(15.6)
	Occasionally	284(25.5)	52(28)	232(25.1)
	Never	586(52.8)	111(59.7)	475(51.5)
Attend religious institution	Regularly	344(31)	34(18.3)	310(33.6)
	1–2 times in 2 wks	553(49.9)	79(42.5)	474(51.4)
	Occasionally	116(10.4)	45(24.2)	71(7.7)
	Never	96(8.7)	28(15.1)	68(7.4)
Attending night club	Yes	234(21.1)	113(60.8)	121(13.1)
	No	875(78.9)	73(39.2)	802(86.9)
Watching pornographic movies	Yes	579(52.2)	154(82.8)	425(46)
	No	530(47.8)	32(17.2)	498(54)

### Khat chewing practice of the respondents

**Fig. 2:**
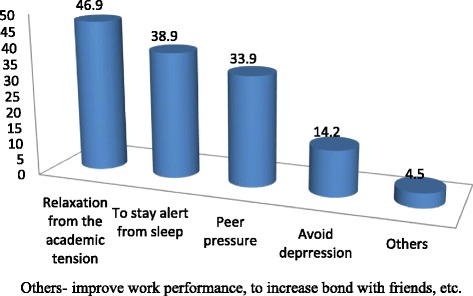
Reasons for khat chewing among college students in Arba Minch Town, March 2015

The lifetime and current prevalence of khat chewing among the participants was 213 (19.2%) and 186 (16.8%) respectively. More than nine in ten (91.1%) of the participants were chewing khat less than 3 times a week. More than half (57.7%) of chewer students started khat chewing during their high school studies. One hundred eighteen (55.4%) of the respondents were long-term (more than 2 years) khat chewers. The reasons mentioned for khat chewing are shown below (Fig. 2).

### Sexual behavior of the respondents

More than four in ten, 466 (42%) respondents had ever practiced sexual intercourse. The age of first sexual intercourse ranges from 14 to 24 years with a mean age of 17.6 ± 1.73 year. Out of the 466 sexually active respondents, 266 (57%) had their first sex before joining the college. The main reasons for their sexual debut were sexual desire 197 (42.4%), peer pressure 143 (30.8%), maintaining good relationship with a partner 61 (13.1%), gaining money 15 (3.2%), passing examinations 26 (5.6%) and for other reasons such as to express love for a partner or to develop sexual experience 22 (4.7%). From the total 1109 respondents, 452 (40.8%) had risky sexual behaviors in their lifetime. Out of 466 sexually active respondents, 307 (65.9%) had multiple sexual partners, majority 409 (89.3%) of respondents used condom inconsistently, 260 (56.9%) started sexual intercourse before the age of 18 and 33 (12.1%) of males had sex with commercial sex workers in their lifetime. Regarding current sexual behavior, 444 (40.1%) of respondents were engaged in sexual intercourse during the last six months. Out of 444 sexually active respondents, 136 (30.6%) had multiple sexual partner, 377 (88.7%) respondents never used condom or used it inconsistently. The reasons mentioned by the respondents for not using condom was trust on partner 171 (40.9%), inaccessibility of condom 126 (30.1%), perceived decrease in sexual pleasure 111 (26.5%), hating condoms 142 (27.7%), embarrassed to buy condom 124 (26.9%), others (fear of side effect, religious prohibition, cultural taboo) 47 (11%). The most common place to obtain condom reported by the respondents was health institution 867 (78.2%) followed by hotels 636 (57.3%). Twelve (4.4%) male students had sex with commercial sex workers in the last six months. More than one-third 405 (36.5%) of the respondents had RSB in the last six month (Table 2).

**Table 2:** Comparison of sexual behavior among khat chewer and non-chewer college students in Arba Minch town, March 2015

Life time sexual behavior		Total	Life time khat chewing		*P*-value
		No (%)	Yes No (%)	No No (%)	
Life time sexual intercourse (*N* = 1109)	Yes	466(42)	178(83.6)	288(32.1)	0.001
	No	643(58)	35(16.4)	608(67.9)	
Age at first sexual intercourse (*N* = 466)	< 18	260(56.9)	94(53.7)	166(58.9)	0.28
	> = 18	197(43.1)	81(46.3)	116(41.1)	
No sexual partner (*N* = 466)	One	159(34.1)	36(20.2)	123(42.7)	0.01
	More than one	307(65.9)	142(79.8)	165(57.3)	
Regular Condom use with non-regular partner (*N* = 458)	Yes	49(10.7)	23(12.9)	26(9.3)	0.34
	No	409(89.3)	155(87.0)	69(90.7)	
Sexual intercourse with CSW (*N* = 272)	Yes	33(12.1)	19(15.2)	14(9.5)	0.12
	No	239(87.9)	106(84.8)	133(90.5)	
Risky sexual behavior (*N* = 1109)	Yes	452(40.8)	175(82.2)	307(30.9)	0.001
	No	759(59.2)	38(27.8)	690(69.1)	
Current sexual behavior			Current khat chewing		
Sex in the last 6 month (*N* = 1109)	Yes	444(40.1)	154(82.8)	290(31.5)	0.001
	No	664(59.9)	32(17.2)	632(68.5)	
Age at first sexual intercourse (*N* = 444)	< 18	16(3.6)	11(7.1)	5(1.7)	0.002
	> = 18	428(96.4)	143(92.9)	285(98.3)	
No of sexual partner (*N* = 444)	One	308(69.4)	82(53.2)	226(77.9)	0.001
	more than one	136(30.6)	72(46.8)	64(22.1)	
Regular Condom use with non- regular partner (*N* = 425)	Regular	48(11.3)	24(16.1)	24(8.7)	0.13
	Never	377(88.7)	125(83.9)	252(91.3)	
Sexual intercourse with CSW (*N* = 260)	Yes	12(4.7)	5(4.4)	7(4.8)	0.67
	No	248(95.3)	109(95.6)	139(95.1)	
Risky sexual behavior (*N* = 1109)	Yes	405 (36.5)	142(76.3)	263(28.5)	0.001
	No	806(63.5)	44(23.7)	762(71.5)	

### Comparison of risky sexual behavior among khat chewer and non-chewer college students in Arba Minch town

Majority 178 (83.6%) of khat chewers and almost one-third 288 (32.1%) of non-chewers ever practiced sexual intercourse. The prevalence of lifetime RSB was 82.2% among khat chewers and 30.9% among non-chewers (*p*-value< 0.001). Majority 154 (82.8%) of current khat chewers and 290 (31.5%) of current non-chewers ever practiced sexual intercourse in the last 6 months. The prevalence of current RSB was 76.3 and 28.5% among current khat chewers and non-chewers respectively (*P*-value< 0.001) (Table 2).

### Factors associated with current risky sexual behavior among college students in Arba Minch town

The odds of current RSB among male respondents was 1.82 times higher than that of females (AOR = 1.82; 95% CI: (1.28–2.6). An increase in a student’s age by one year increases the odds of current risky sexual behavior by 1.18 (AOR = 1.18; 95%CI: 1.09–1.28). Respondents from urban areas had 1.63 times higher odds of current RSB than those from rural areas (AOR = 1.63; 95% CI: 1.17–2.28). Respondents living away from family were found to have 2.45 times (AOR = 2.45 95% CI = 1.62–3.7) higher odds of current RSB than those who were living with their family. Respondents with high peer pressure had 2.58 times (AOR = 2.58; 95%CI: 1.85–3.59) higher odds of current RSB than those who had low peer pressure. An increase in a respondent’s average grade by one point decreased the odds of current RSB by 0.98(AOR 0.98; 95% CI: 0.96–0.99).

Those respondents who attended religious institutions regularly had 0.24 times (AOR =0.24; 95%CI: .12–0.42) lower odds of current RSB than those who never attended a religious institution. Respondents who watched pornographic movies had 2.5 times (AOR = 2.51 95% CI = 1.79–3.51) higher odds of current RSB than those who never watched pornographic movies. Students who chewed khat had more than three times higher odds of current RSB than those who did not chew chat (AOR = 3.02, 95% CI: 1.91–4.76). Similarly, those who drank alcohol had more than two times higher odds of current RSB (AOR = 2.26, 95% CI = 1.54–3.35) than those who didn’t drink alcohol (Table 3).

**Table 3:** Factors associated with current risky sexual behavior among college students in Arba Minch Town, March 2015

Variables		Current RSB		COR (95% CI)	AOR(95% C.I)
		Yes	No		
		No (%)	No (%)		
Age ©		405 (36.5)	704 (63.5)	1.17(1.10–1.25)	1.18(1.09–1.28)*
Sex	Male	236(40.2)	351(59.8)	1.40(1.10–1.80)	1.82(1.28–2.6)*
	Female	169(32.4)	353(67.6)	1	1
Previous residence	Urban	245(45.4)	295(54.6)	2.12(1.66–2.72)	1.63(1.17–2.28)*
	Rural	160(28.1)	409(71.9)	1	1
Living arrangement	Away from family	333(39)	519(61)	1.65(1.22–2.24)	2.45(1.62–3.7)*
	With family	72(28)	185(72)	1	1
Peer pressure	High	116(19.6)	477(80.4)	5.24(4.01–6.84)	2.58(1.85–3.59)*
	Low	289(56)	227(44)	1	1
Father educational status	No formal education	87(39.4)	134(60.6)	0.93(0.67–1.29)	1.48(.96–2.31)
	Primary	119(29.5)	285(70.5)	0.60(0.45–0.79)	0.74(0.51–1.07)
	Secondary and above	199(41.1)	285(58.9)	1	1
Type of college	Private	73(40.8)	106(59.2)	1.24(0.90–1.72)	0.91(0.59–1.42)
	Public	332(35.7)	598(64.3)	1	1
Year of study	Year three	132(45.7)	157(54.3)	1.81(1.34–2.45)	1.270(.85–1.9)
	Year two	126(35.3)	231(64.7)	1.73(0.88–1.57)	1.06(0.74–1.54)
	Year one	147(31.7)	316(68.3)	1	1
Average grade©	Average grade	405(36.5)	704(63.5)	0.06(0.98–1.00)	0.98(0.96–0.99)*
Attending religious institution	Regularly	69(20.1)	275(79.9)	0.78(0.11–0.29)	0.23(0.12–0.42)*
	Often	211(38.2)	342(61.8)	0.4(0.28–0.69)	0.59(0.34–1.05)
	Occasionally	69(59.5)	47(40.5)	1.05(0.61–1.82)	0.81(0.38–1.65)
	Never	56(58.3)	40(41.7)	1	1
Attending nightclub	Yes	152(65)	82(35)	4.56(3.36–6.19)	1.42(0.93–2.16)
	No	252(28.8)	623(71.2)	1	1
Watching pornographic movies	Yes	307(53)	272(47)	4.98(3.76–6.54)	2.51(1.79–3.51)*
	No	89(17)	432(83)	1	1
khat chewing	Yes	142(76.4)	44(23.7)	7.55(5.37–10.6)	3.02(1.91–4.76)*
	No	263(28.5)	660(71.6)	1	1
Alcohol drinking	Yes	214(66.3)	109(33.7)	6.12(4.61–8.11)	2.26(1.54–3.35)*
	No	191(24.3)	595(75.7)	1	1
Cigarette smoking	Yes	46(88.5)	6(11.5)	14.91(6.31–35.23)	2.26(0.83–6.16)
	No	359(44)	698(66)	1	1

© continous variables *statistically significant association at *p*-value < 0.05

## Discussion

The prevalence of lifetime and current RSB was found to be 40.7 and 36.5% respectively. The finding was in line with a study conducted among preparatory students in Jimma Zone where the lifetime RSB was 42.1% [28]. However, it was higher than studies conducted among high school students in Humera and Gondar, North West Ethiopia where lifetime RSB were 13.7 and 25% respectively. This difference might be due to the majority of students were less than 18 years in the previous studies [13, 31]. Thus, when one gets older, peer pressure and social acceptance for sexual intercourse also increases. It could also be owing to the fact that majority of the students in high schools were living with their families which was a protective factor for RSB.

The prevalence of lifetime and current RSB among khat chewers was 82.2 and 74.2% respectively. This was significantly higher than that of non-chewers (30.9 and 27.6%) respectively. This finding was in line with studies conducted in Jimma University, South West Ethiopia, Bahir Dar and Humera, North West Ethiopia where RSB among chewers (51, 61.1 and 52.5%) was significantly higher than non-chewers (19.8, 9.3 and 16.5%) respectively [6, 12, 13]. The reason might be during the hypo-manic phase; khat chewers might not be capable of rational judgment and not be able to predict the serious consequences of their actions. Thus, they might be engaged in sexual intercourse. However, the finding showed difference with the Haramaya University study where RSB among khat chewers (68.4%) was slightly higher than non-chewers (62.7%) [14]. The difference might be due to the currently increased efforts by the promotion of behavioral change communication (BCC) through youth friendly services (YFS) in universities with the aim of increasing risk perception and condom distribution activities. This has an implication for expansion of YFS to student communities in colleges in alignment with the Global Strategy for Women’s, Children’s and Adolescents’ Health (GSWCAH)(2016–2030) and the HSTP (2016–2020) of Ethiopia for attainment of their efforts in ensuring universal access to sexual and reproductive health-care services [32, 33].

Khat chewing was found to be an independent factor for RSB. Respondents who chewed khat had higher odds of RSB than those who did not chew chat. Similarly, those who drank alcohol had higher odds of RSB than those who did not drink alcohol. The finding was in line with studies from Yemen and Ethiopia [12, 14]. The reason might be that students are initiated for sexual intercourse after having substances and might be due to the nature of substance in decreasing inhibitions, altering rational decision-making and increasing risk-taking behaviors. Besides, alcohol limits the cognitive capacity of individuals that leads them to have unsafe sex. This implies that BCC should be strengthened among college adolescents/youths on substance use and its consequences together with regulatory mechanisms in the use of substances. However, the finding was not consistent with studies conducted in Jimma and Bahir Dar that revealed no association between khat chewing and RSB [6, 28]. Similarly, findings from Humera and Addis Ababa revealed that there was no association between smoking cigarette, khat chewing and alcohol drinking and RSB. The reason might be due to inadequate chewers to compare the groups in the previous studies [7, 13].

The study showed that male respondents had higher odds of RSB than females. This finding was consistent with the Humera and Haramaya studies [13, 14]. This could be explained by the Ethiopian culture that favors males for initiation of sex, access to resources and decision making which might have forced them to have a relatively higher risky sexual practice. This has an implication that there is a need to break the culture through promotion of sexuality education with particular emphasis on safe sex practices among male students in colleges. Higher age groups of respondents’ showed a positive association with RSB. This was in agreement with findings from previous literatures. The possible explanation could be the fact that as age increases, involving in sexual practice increases due to the social acceptance of sexual intercourse in late age [12, 18, 34].

Respondents who were living away from their family had higher odds of RSB than those who were living with their family. This finding was consistent with previous studies [6, 13, 18, 29]. The reason might be that students who were living in rental house away from family members might spend most of their time with their intimate friends that in turn results in sharing RSBs because of peer pressure. The other possible reason could be freedom from family control by itself creates a room to initiate sexual intercourse. This has an implication for expansion of formal training on life skills among college students where the majorities live in rental houses in the general community. Respondents from urban areas had higher odds of RSB than those from rural area. The possible explanation could be due to cultural taboos of sexual relationships in rural areas of the country do have a protective effect against sexual initiation and acquiring RSB unlike urban youths having a better freedom in life styles and decision-making power [30]. On the other side, it could also be due to the fact that urban residents have a better confidence in providing genuine information than rural residents as students from rural area are ashamed of reporting on sexual matters. The finding was inconsistent with a study conducted in Jimma, South West Ethiopia [30] where there was no association between residence and RSB.

The study further found that students who report having high peer pressure had higher odds of RSB as compared to those who had low peer pressure. This finding was in line with other previous studies [34, 35]. This might be due to the fact that such students were at higher probability of sharing information regarding their day to day life and in a position to conform to peers’ informal rules. Strengthening school peer education programmes on safe sexual practices could avert this situation [35].

An increase in average grade by one point decreased the odds of risky sexual behavior by 0.98 (AOR = 0.98; 95% CI: 0.96–0.99). The reason might be that academically poor students practice sexual intercourse with teachers and their colleagues to pass examinations. The other possible explanation might be students with poor academic performance cannot focus on their academic affairs and hence would be exposed to watching pornographic movies and as a result they could experience RSB. Tailoring sexuality education to students with poor academic performance is critical.

Those respondents who regularly attended religious institution had lower odds of RSB than those who had never attended religious institutions. This finding was similar with studies conducted in Jimma, Humera, and Haramaya [13, 14, 30]. The reason might be an individual who attends religious institution regularly might stick to the doctrines that do not support sexual practice out of marital union and hence abstain from pre-marital sex or do it cautiously. Respondents who watched pornographic movies had higher odds of RSB than those who never watched pornographic movies. This finding was in agreement with some previous studies [7, 13]. The reason might be the fact that watching such movies might increase sexual desire and urge them to rush for sexual intercourse without considering its consequences. Hence, this implies that students’ viewing of pornography should be considered in sexuality educations because of the growing utilization of internet through smartphones and exposure to social media [36].

Involving students in anti-AIDS club is important to change the attitude and practice of RSB that enables students to have information on safe sex. All public colleges had an anti-AIDS club in their compound. However, in this study and a previous study [29], presence of anti-AIDS clubs and membership did not show statistically significant association with RSB. Thus, the function and effectiveness of anti-AIDS clubs needs further investigation.

The study has few limitations and caution must be exercised in generalizing the result of this study. Firstly, it is cross-sectional in nature and may not explain the temporal relationship between the outcome variable and some explanatory variables. Secondly, the study topic by its nature assesses personal and sensitive issues related to sexuality which might have caused underreporting of some behaviors. Thirdly, due to the quantitative nature of the study, there were no event-level measurements of risky sexual behavior; the studied association may be due to some unmeasured risk factors. Fourthly, since RSB and khat chewing were measured over the last 6 months and even beyond that retrospectively, there could be a chance of recall bias to the reported data.

## Conclusions

Considerable proportions of students were engaged in khat chewing and RSB. The prevalence of RSB among khat chewers was significantly higher than non-chewers. Khat chewing, alcohol drinking, male sex, older age, urban residence, living away from family, high peer pressure, poor academic performance, not attending religious institution, watching pornographic movies were found to be factors having significant association with current RSB. Therefore, this study has explicitly showed that khat chewing was found to be an independent factor affecting RSB in a comparative manner on a large sample of college adolescents/youths in Southern Ethiopia by measuring RSB in a relatively inclusive way. However, as this evidence varies in different settings across the country, there is a need for further research using other strong designs.

The ministry of health, regional and zonal health bureaus and other non-governmental partners should strengthen their support to colleges by considering the identified factors for policy and programme improvements in their efforts to have healthy adolescents and by extension healthy citizens. Hence, policy makers and programme planners working on the promotion of adolescent and youth reproductive health should strengthen their efforts on behavioral change communication by establishing YFSs in college settings with particular emphasis on risk perception and life skills training. Peer to peer sexuality education should target students with high order ages, who are male, living away from their families, urban residence, and substance (khat or alcohol) users.

## Additional file

Additional file 1: The “additional file” document is a raw data file in the form of excel spreadsheet that has been exported from the SPSS data used in the analysis of this study. (XLS 1002 kb)

## Abbreviations

AIDS: Acquired immuno deficiency virus
AOR: Adjusted odds ratio
BCC: Behavioural change communication
CI: Confidence interval
COR: Crude odds ratio
GSWCAH: Global strategy for women’s, children’s and adolescents’ health
HIV: Human Immuno Virus
HSTP: Health sector transformation plan
ID: Identification
RSB: Risky sexual behaviour
SPSS: Statistical package for social sciences
STI: Sexually Transmitted Infections
TVET: Technical and Vocational Education and Training
VIF: Variance inflation factor
YFS: Youth friendly service
